# Dietary sesame seed supplementation enhances productive performance, antioxidant status, serum lipids, gut morphology, and pathogen load in Rhode Island Red laying hens

**DOI:** 10.1016/j.psj.2026.106618

**Published:** 2026-02-11

**Authors:** Saba Ajam, Sar Zamin Khan, Hanan Al-Khalaifah, Saher Musrrat, Rifat Ullah Khan, Abdul Hafeez Abdul Razzaq, Ala Abudabos, Ihteshamul Haq, Ibrahim A. Alhidary

**Affiliations:** a Department of Poultry Science, Faculty of Animal Husbandry and Veterinary Sciences, The University of Agriculture, Peshawar, Pakistan; b Environment and Life Sciences Research Center, Kuwait Institute for Scientific Research, Safat, Kuwait; c Department of Bioinformatics, Quaid-e-Azam university, Islamabad, Pakistan; d Physiology Lab, College of Veterinary Sciences, Department of Animal Husbandry and Veterinary Sciences, The University of Agriculture, Peshawar, Pakistan; e Agriculture Development Company- LTD, CR 1010112444, Riyadh 12341, Saudi Arabia; f Departent of Food and Animal Sciences, College of Agriculture, Tennessee State University, Nashville, TN 37209, USA; g Institute of Biotechnology and Genetic Engineering, The University of Agriculture, Peshawar, Pakistan; h Department of Animal Production, College of Food and Agriculture Science, King Saud University, Riyadh, Saudi Arabia

**Keywords:** Sesame seed supplementation, Laying hens, Antioxidant status, Intestinal morphology, Serum lipid profile

## Abstract

This study investigated the effects of supplementation of dietary sesame seed (SS) on the performance, egg quality, serum biochemical profile, intestinal morphology, and microbial load of Rhode Island Red laying hens. A total of 240 Rhode Island Red hens (30 weeks old) were assigned to four dietary treatments in a completely randomized design for fifteen weeks, where sesame seed was incorporated at 0, 10, 15, or 20 g/kg in isonitrogenous diets. Performance parameters were recorded daily, egg quality was assessed biweekly, and blood, fecal, and intestinal samples were collected at the end of the trial for biochemical, microbiological, and histomorphological analyses. Partial substitution of soybean meal with sesame seed significantly (*P* < 0.05) improved daily and weekly egg production, hen-day egg production, and feed conversion ratio, with the highest replacement level (20 g/kg) yielding the greatest enhancement. Egg quality characteristics were not influenced by dietary treatment. The serum lipid profile improved significantly (*P* < 0.05), as sesame-based replacement reduced total and LDL cholesterol while increasing HDL cholesterol. Antioxidant status was also enhanced (*P* < 0.05), as indicated by increased SOD, GPx, and total antioxidant capacity, although MDA levels remained unaffected. Furthermore, soybean meal replacement with sesame seed reduced (*P* < 0.05) fecal *Salmonella* and *Escherichia coli* counts and improved (*P* < 0.05) duodenal morphology, reflected by shallower crypt depths and higher villus-to-crypt ratios. Overall, sesame seed up to 20 g/kg enhanced productivity, antioxidant defense, lipid metabolism and pathogen reduction without compromising egg quality, highlighting sesame seed as a viable alternative protein source in layer diets.

## Introduction

The increasing global demand for safe, affordable, and high-quality poultry products has intensified interest in sustainable feed strategies that enhance production efficiency while improving bird health and product quality (Abudabos et al., 2013; Alqahtani et al., 2024; Siddiqui et al., 2025; Mba et al., 2025). In this context, considerable attention has been directed toward functional feed ingredients derived from oilseeds and medicinal plants, not necessarily as complete protein substitutes, but as **nutritional supplements capable of improving physiological resilience and gut health** in poultry (Al-Suwailem et al., 2024; Hailat et al., 2024; Nguyen et al., 2024; Khan et al., 2025). Such ingredients are particularly valuable in reducing dependence on synthetic growth promoters and supporting antibiotic-free production systems (Almahallawi et al., 2024, Nouri et al., 2025).

Sesame (*Sesamum indicum* L.) seed is recognized for its rich nutritional and functional profile, containing moderate levels of digestible protein, high oil content (50–60 %), essential minerals, and biologically active lignans such as sesamin, sesamol, and sesamolin (Abbas et al., 2022; Sharma et al., 2020). While sesame seed protein alone may not fully replace soybean meal at low dietary inclusion levels, **its bioactive constituents can exert pronounced antioxidant, lipid-modulating, and antimicrobial effects**, even when incorporated in small amounts (Yaseen et al., 2021; Salavati et al., 2021).

Oxidative stress and dyslipidemia are major physiological challenges in laying hens, particularly during peak production. Dietary sesame-derived lignans have been shown to enhance antioxidant enzyme activity, reduce lipid peroxidation, and improve serum lipid profiles, thereby contributing to improved metabolic health and egg production efficiency (Salavati et al., 2021). These functional attributes make sesame seed a promising **nutraceutical feed additive**, rather than a primary protein replacer, in layer nutrition.

Maintenance of gut integrity and microbial balance is equally critical for nutrient utilization, immune competence, and disease resistance in laying hens (Rahimian et al., 2024). Sesame seed possesses antimicrobial properties that may help suppress enteric pathogens such as *Salmonella* and *Escherichia coli*, while supporting intestinal morphology and mucosal integrity (Telmadarreh et al., 2025). Such effects are particularly relevant under commercial production conditions where subclinical infections can impair performance.

Despite these advantages, studies evaluating **low-level dietary inclusion of sesame seed as a functional supplement** in laying hens remain limited, and reported outcomes vary depending on inclusion rate, diet formulation, and bird genotype (Reuben et al., 2025). Therefore, controlled evaluations are needed to clarify its biological effects independent of major protein replacement.

Accordingly, the present study hypothesized that **graded dietary supplementation of sesame seed**, without substantial replacement of soybean meal, would improve productive performance, antioxidant status, serum lipid profile, gut morphology, and pathogen load in Rhode Island Red laying hens in a dose-dependent manner.

## Materials and methods

### Experimental design and birds

The study was conducted to evaluate the effects of graded dietary supplementation of sesame seed (SS) on productive performance, egg quality, serum biochemical parameters, intestinal morphology, and microbial profile of laying hens. A total of 240 healthy, 30-week-old Rhode Island Red (RIR W-30) laying hens were used in the experiment. Birds were randomly allocated to four dietary treatments following a completely randomized design (CRD), with five replicates per treatment. Each replicate consisted of a cage housing 12 birds, and the cage was considered the experimental unit for all performance parameters, including feed intake, egg production, feed conversion ratio, and hen-day egg production. The experiment was conducted over a 15-week period.

The birds were housed in semi-controlled cages (dimensions: 50 × 40 × 40 cm per cage) with wire mesh flooring. Environmental conditions, including temperature (24 ± 2 °C) and relative humidity (55–65 %), were maintained according to standard management guidelines for layers. A 16 h light and 8 h dark photoperiod was applied throughout the trial using automated lighting. Feed (110 g/bird/day) and water were provided ad libitum. Daily health observations were recorded to monitor any signs of disease, stress, or abnormal behavior. All procedures were conducted following institutional ethical standards for animal care and welfare.

### Dietary treatments

Four experimental diets were formulated to evaluate the effects of graded dietary inclusion of sesame seed (SS) in laying hen diets. The treatments consisted of a control diet without sesame seed supplementation (0 g/kg SS) and three experimental diets supplemented with sesame seed at levels of 10, 15, and 20 g/kg of feed, respectively. These inclusion levels were selected to assess the functional effects of sesame seed supplementation without substantially replacing the primary protein source in the diet.

All basal diets were formulated to meet or exceed the nutrient requirements for laying hens as recommended by the National Research Council (NRC, 1994) (Table 1). To ensure consistency among treatments, diets were formulated to be isocaloric and isonitrogenous, with adjustments made to other ingredients as required.

**Table 1:** Ingredient composition and nutrient specification of experimental diets fed to laying hens (% as-fed).

Ingredient	Basal diet
Yellow corn (maize)	55.0
Soybean meal (44 % CP)	22.0
Sesame seed	0.0
Limestone (fine)	8.5
Limestone (coarse)	6.0
Dicalcium phosphate	1.6
Vegetable oil	3.0
Salt	0.35
DL-Methionine	0.20
L-Lysine HCl	0.05
Vitamin–mineral premix¹	0.50
Total	100
Nutrient specification (calculated)	
Nutrient	Control
Metabolizable energy (kcal/kg)	2750
Crude protein (%)	17.5
Digestible lysine (%)	0.85
Digestible methionine + cystine (%)	0.75
Available phosphorus (%)	0.40
Calcium (%)	4.00
Sodium (%)	0.16

Experimental diets were formulated by incorporating sesame seed into the basal diet at levels of 10, 15, and 20 g/kg feed. Sesame seed inclusion was achieved by proportionally adjusting (w/w) soybean meal and yellow corn to maintain isocaloric and isonitrogenous diets across treatments. ¹Premix supplied per kg of diet (minimum): vitamin A 10,000 IU; vitamin D₃ 3,000 IU; vitamin E 30 mg; vitamin K 3 mg; B-complex vitamins; Mn 60 mg; Zn 60 mg; Cu 8 mg; I 1 mg; Se 0.3 mg.

Prior to dietary incorporation, sesame seeds were thoroughly cleaned to remove impurities, dehulled, and finely ground to facilitate homogenous mixing and improve nutrient availability. The processed sesame seed was then incorporated into the basal diet according to the designated treatment levels using a mechanical mixer to ensure uniform distribution. Experimental diets were prepared on a weekly basis and stored in airtight containers under dry conditions to preserve feed quality and prevent oxidative deterioration.

Birds were provided ad libitum access to fresh drinking water, while feed was offered twice daily, in the morning and afternoon, to minimize feed wastage and ensure consistent intake throughout the experimental period.

### Chemical composition of sesame seed and diet formulation

The SS used in the experimental diets was chemically analyzed prior to diet formulation, and its composition is presented in Table 2. On a dry matter basis, sesame seed contained high levels of ether extract along with moderate crude protein, crude fiber, and mineral contents, confirming its suitability as an energy- and bioactive-rich feed ingredient. The peroxide value of sesame seed oil was within acceptable limits, indicating good oxidative stability. In addition, sesame seed lipids were rich in unsaturated fatty acids, mainly oleic (C18:1) and linoleic (C18:2) acids, and contained antioxidant lignans (sesamin and sesamolin).

**Table 2:** Chemical composition of sesame seed used in the experiment (DM basis).

Component	Value
Dry matter (DM, %)	94.2
Crude protein (CP, %)	21.4
Ether extract (EE, %)	50.6
Crude fiber (CF, %)	6.1
Ash (%)	5.2
Calcium (Ca, %)	1.08
Phosphorus (P, %)	0.67
Peroxide value (meq O₂/kg oil)	3.2
Oleic acid (C18:1, %)	39.5
Linoleic acid (C18:2, %)	42.8
Palmitic acid (C16:0, %)	9.4
Stearic acid (C18:0, %)	5.6
Sesamin + sesamolin (mg/kg)	8,500

Experimental diets were formulated to be iso-nitrogenous and iso-caloric, with graded inclusion of sesame seed at 0, 10, 15 and 20 g/kg. Ingredient proportions were adjusted to maintain comparable levels of crude protein, metabolizable energy, and major nutrients across all treatments in accordance with the nutrient requirements of laying hens. Chemical analysis of the experimental diets confirmed that incremental supplementation of sesame seed from 0 to 20 g/kg did not result in significant differences in the final nutritional quality of the diets, including crude protein and energy content. Therefore, any observed treatment effects can be attributed to the functional properties of sesame seed rather than variations in dietary nutrient composition.

### Performance parameters

Feed intake was measured daily at the replicate level by recording feed offered and feed remaining at the end of each day. Egg production was recorded daily for each replicate, and eggs were weighed individually to calculate average egg weight. Hen-day egg production (%) was calculated weekly as:

Hen-day production (%) = (Number of eggs produced / (Number of hens alive × 7)) × 100

Eggs were collected and counted daily from each replicate group, and the number of live hens was recorded concurrently to account for any mortality. Daily egg production values were then averaged over the experimental period (or weekly intervals) to obtain mean egg production per treatment group.

Feed conversion ratio (FCR) was calculated as the amount of feed consumed per dozen eggs produced:

FCR=Feedintake(g)/Dozeneggsproduced

Egg quality was evaluated at weeks 4 and 8 of the experiment. From each replicate, three eggs were randomly selected for assessment to ensure representative sampling. The parameters measured included shell thickness, yolk weight, albumen height, and Haugh Unit. Shell thickness was determined at three distinct points on each egg—the blunt end, the middle section, and the pointed end—using a precision micrometer (Mitutoyo, Japan) to obtain an average thickness value for each egg. Yolk weight was measured using a digital analytical balance with a precision of ±0.01 g to ensure accurate determination of the yolk component. Albumen height was recorded using an albumen gauge, which provided a measure of the thick albumen quality. The Haugh Unit (HU), an index widely used to evaluate internal egg quality, was calculated according to the formula described by Silversides and Budgell (2004):

$$\text{HU}\;=100\times \text{log}\;\left(H\;+\;7.57\;-\;1.7\;W^{\wedge }0.37\right)$$

where H represents the albumen height in millimeters and W denotes the egg weight in grams. This method allowed the assessment of both the structural integrity of the eggshell and the internal quality of the egg components, providing a comprehensive measure of egg quality in response to dietary treatments. All measurements were performed in triplicate and averaged at the replicate level to minimize variability and ensure reliable data for statistical analysis.

### Blood sampling and serum analysis

At the end of the experiment, two birds from each replicate were randomly selected for blood collection. Approximately 3–5 mL of blood was drawn from the wing vein using sterile disposable syringes and transferred into plain vacutainer tubes. The samples were allowed to clot at room temperature (22–25°C) for 30–40 min and subsequently centrifuged at 3,000 × *g* for 10 min at 4°C to obtain clear serum. The serum fraction was carefully aspirated and stored at −20°C until analysis. For blood biochemical analysis, two birds were randomly selected from each replicate cage at the end of the experimental period. Samples obtained from birds within the same cage were averaged prior to statistical analysis, and the replicate cage remained the experimental unit for these measurements. This approach ensured consistency of the experimental unit across all analyses and avoided pseudo-replication.

Serum lipid profile, including total cholesterol, high-density lipoprotein (HDL), low-density lipoprotein (LDL), and triglycerides, was determined using commercial colorimetric assay kits (Randox Laboratories Ltd., Crumlin, UK) following the manufacturer’s instructions. Absorbance values for all lipid parameters were measured using a UV–visible spectrophotometer (Shimadzu UV-1800, Japan), and concentrations were calculated based on standard calibration curves supplied with the kits. Antioxidant status was evaluated by measuring the activities or concentrations of superoxide dismutase (SOD), glutathione peroxidase (GPx), total antioxidant capacity (TAC), and malondialdehyde (MDA). These assays were performed using enzyme-linked immunosorbent assay (ELISA) kits (Nanjing Jiancheng Bioengineering Institute, China). All ELISA plates were read at their respective wavelengths using a microplate reader (BioTek ELx808, Winooski, USA).

### Microbial load analysis

Fresh fecal samples were collected from two birds per each replicate during the final week of the experiment using sterile spatulas and placed into labeled sterile containers. Approximately 1 g of fecal material from each sample was homogenized in 9 mL of sterile buffered peptone water (BPW) to obtain a 10⁻¹ dilution. Ten-fold serial dilutions were subsequently prepared up to 10⁻⁶ using sterile BPW, following standard microbiological procedures. For Salmonella spp., detection and enumeration followed ISO 6579:2017 guidelines. Briefly, pre-enrichment was performed by incubating fecal homogenates in BPW at 37°C for 18–24 h. Aliquots were then transferred to Rappaport–Vassiliadis (RV) selective enrichment broth and incubated at 41.5°C for 24 h. Enriched cultures were streaked onto Xylose Lysine Deoxycholate (XLD) agar and incubated at 37°C for 24 h. Presumptive Salmonella colonies were identified based on characteristic morphology (red colonies with black centers) and were further confirmed using biochemical profiling (API 20E, bioMérieux, France) and slide agglutination serology. For Escherichia coli, enumeration followed ISO 16649-2:2001 procedures. Appropriate dilutions were surface-plated onto MacConkey agar and incubated aerobically at 37°C for 24 h. Typical lactose-fermenting colonies (pink to red colonies) were counted, and results were expressed as colony-forming units per gram (CFU/g) of fresh feces. Only plates with 30–300 colonies were considered for enumeration. Final bacterial counts were calculated by multiplying the observed colony numbers by the corresponding dilution factor and expressed as Log_10_ CFU/g of fecal material.

### Intestinal morphology

At the end of the experiment, two birds from each replicate were humanely euthanized for intestinal tissue collection. Immediately after euthanasia, the abdominal cavity was opened, and segments of the duodenum and jejunum (approximately 2 cm in length) were excised from the midpoint of each region. The samples were gently flushed with cold phosphate-buffered saline (PBS; pH 7.4) to remove digesta without damaging the mucosa and then immersed in 10 % neutral buffered formalin for fixation. Tissues were fixed for at least 24 h at room temperature to ensure adequate penetration. Following fixation, samples were processed using a standard histological protocol. Briefly, tissues were dehydrated through an ascending ethanol series (70, 80, 90, 95, and 100 %), cleared in xylene, and embedded in paraffin blocks. Serial sections of 5 µm thickness were prepared using a rotary microtome (Leica RM2125, Germany). The sections were mounted on glass slides, dried, and stained with hematoxylin and eosin (H&E) to visualize mucosal architecture. Histological slides were examined under a light microscope (Olympus CX23, Japan) at 40 × magnification. Villus height (measured from the villus tip to the villus-crypt junction) and crypt depth (measured from the villus-crypt junction to the base of the crypt) were determined using ImageJ software (National Institutes of Health, USA). For each intestinal region, at least 10 well-oriented, intact villi and their associated crypts were measured per sample to obtain replicate means. The villus height–to–crypt depth ratio (VH:CD) was calculated to provide an additional index of mucosal integrity.

### Statistical analysis

All experimental data were analyzed using one-way analysis of variance (ANOVA) to evaluate the effects of dietary sesame seed supplementation. Statistical analyses were performed using SPSS software (Version 26.0; IBM Corp., Armonk, NY, USA). Performance parameters, including feed intake, egg production, feed conversion ratio (FCR), and hen-day egg production, were recorded weekly throughout the 15-week experimental period. For statistical analysis, weekly measurements were averaged over the entire experimental period so that each replicate contributed a single independent observation per treatment. Accordingly, treatment effects on performance parameters were assessed using one-way ANOVA. Egg quality traits, which were measured at specific sampling points, were also analyzed using one-way ANOVA. When significant treatment effects were detected, differences among treatment means were compared using Tukey’s multiple comparison test. Statistical significance was declared at *p* < 0.05. All results are presented as means ± standard error of the mean (SEM). Prior to analysis, data were examined for normality and homogeneity of variances, and all datasets met the assumptions required for parametric analysis; therefore, no data transformation was necessary.

## Results

The effects of graded dietary supplementation of sesame seed on production performance are presented in Table 3. Daily feed intake differed significantly among treatments (*p* = 0.04), with hens in the SS-10 group exhibiting a marginally higher intake than the control, while SS-15 and SS-20 did not differ from the control. Both daily and weekly egg production improved significantly with increasing levels of sesame seed inclusion (*p* = 0.03 and *p* < 0.001, respectively). The SS-15 and SS-20 groups showed superior performance compared with the control and SS-10 groups. Feed conversion ratio (per dozen eggs) was significantly improved in all sesame-supplemented groups (*p* < 0.001), with a progressive improvement. Similarly, hen-day egg production increased significantly in response to sesame seed inclusion (*p* < 0.001), with the highest response recorded in the SS-20 group.

**Table 3:** Impact of Seasame seed supplemental diets on production parameters (mean ± SEM) of laying hens (*n* = 5).

Parameters	SS-0	SS-10	SS-15	SS-20	P values
Daily Feed intake (g)	103.8 ± 0.44^ab^	104.26 ± 0.98^a^	103.9 ± 0.32^ab^	103.6 ± 0.97^ab^	0.04
Egg Production Eggs/day	20.9 ± 0.98^c^	21.72 ± 0.96^b^	22.01 ± 0.03^a^	22.1 ± 0.09^a^	0.03
Egg Production Eggs/week	146.7 ± 0.44^c^	152.04 ± 0.98^b^	154.07 ± 0.56^a^	154.9 ± 0.98^a^	0.01
FCR/Dozen eggs)	1.78 ± 0.03^a^	1.72 ± 0.01^b^	1.69 ± 0.04^c^	1.68 ± 0.01^d^	0.01
Hen day Prod (%)	69.8 ± 1.66^c^	72.4 ± 1.61^b^	73.3 ± 1.48^ab^	73.7 ± 1.97^a^	0.01

Small letters (^a-c^) on means in rows indicate significant difference at *P* < 0.05.

SS-0 (control), SS-10, SS-15 and Ss-20: Sesame seeds were supplemented at the rate of 10, 15 and 20 g/kg.

The effects of dietary treatments on egg quality characteristics are summarized in Table 4. None of the evaluated parameters, including egg weight, shell thickness, shell weight, yolk weight, albumen height, or Haugh unit, were significantly influenced with sesame seed (*p* > 0.05). These results indicate that sesame seed inclusion up to 20 g/kg had no adverse or beneficial effects on either external or internal egg quality attributes.

**Table 4:** Impact of Sesame seed supplemental diets on the egg quality parameters (mean ± SEM) of laying hens (*n* = 5).

Parameters	SS-0	SS-10	SS-15	SS-20	P values
Egg weight (g)	50.22 ± 0.23	51.82 ± 0.11	50.79 ± 0.13	53.60 ± 0.12	0.14
Egg shell thickness (mm)	0.340 ± 0.01	0.345 ± 0.01	0.351 ± 0.01	0.343 ± 0.01	0.63
Egg shell weight(g)	5.28 ± 0.21	5.27 ± 0.32	5.36 ± 0.14	5.42 ± 0.16	0.92
Yolk Weight (g)	16.71 ± 2.65	17.02 ± 2.76	16.28 ± 2.34	16.07 ± 2.43	0.43
Albumin Height (mm)	7.62 ± 0.04	7.66 ± 0.01	7.74 ± 0.05	7.72 ± 0.08	0.52
Haugh Unit	73.48 ± 4.3	73.53 ± 3.6	73.56 ± 5.7	74.12 ± 2.8	0.64

SS-0 (control), SS-10, SS-15 and Ss-20: Sesame seeds were supplemented at the rate of 10, 15 and 20 g/kg.

Serum biochemical responses to sesame seed inclusion are shown in Table 5. LDL cholesterol concentrations were significantly reduced in all supplemented groups compared with the control (*p* = 0.02), with the greatest reduction observed in the SS-20 group. In contrast, HDL cholesterol levels increased significantly with supplementation (*p* = 0.01), again showing the highest values in the SS-20 group. Total cholesterol levels were significantly lower in sesame-supplemented hens compared with the control (*p* < 0.001). Triglyceride concentrations did not differ significantly among dietary treatments (*p* > 0.05).

**Table 5:** Impact of sesame seed supplemental diets on serum biochemical parameters (mean ± SEM) of laying hens (*n* = 5).

Parameters (mg/dl)	SS-0	SS-10	SS-15	SS-20	P value
Cholesterol-LDL	37.52 ± 0.8^a^	36.82 ± 0.5^ab^	36.20 ± 0.4^ab^	35.28 ± 0.5^b^	0.02
Cholesterol-HDL	25.92 ± 0.23^b^	27.12 ± 0.6^ab^	27.63 ± 0.5^ab^	28.23 ± 0.2^a^	0.01
Triglycerides	127.8 ± 0.95	127.8 ± 0.96	127.14 ± 0.89	127.7 ± 0.67	0.42
Total Cholesterol	98.12 ± 0.7^a^	97.32 ± 0.5^ab^	97.12 ± 0.6^ab^	96.08 ± 0.7^b^	0.01

Small letters (^a-b^) on means in rows indicate significant difference at *P* < 0.05.

SS-0 (control), SS-10, SS-15 and Ss-20: Sesame seeds were supplemented at the rate of 10, 15 and 20 g/kg.

Antioxidant enzyme activities and oxidative status indicators are presented in Table 6. Superoxide dismutase (SOD) activity increased significantly in response to sesame seed inclusion (*p* = 0.02), with the highest activity recorded in the SS-20 group. Glutathione peroxidase (GPx) activity also increased significantly across supplemented groups (*p* = 0.01), demonstrating a clear dose-dependent response. Total antioxidant capacity was significantly enhanced with increasing levels of sesame seed supplementation (*p* < 0.001). However, malondialdehyde (MDA) concentrations did not differ significantly among treatments (*p* > 0.05), indicating that lipid peroxidation levels remained relatively stable during the experimental period.

**Table 6:** Impact of Seasame seed supplemental diets on antioxidant parameters (mean ± SEM) of laying hens (*n* = 5).

Parameters	SS-0	SS-10	SS-15	SS-20	P value
SOD (U/mL)	149.71 ± 2.3^b^	150.23 ± 1.5^ab^	150.82 ± 1.6^ab^	152.97 ± 9.7^a^	0.02
GPx (U/mL)	812.32 ± 5.6^d^	815.20 ± 4.2^c^	817.12 ± 3.6^b^	819.03 ± 7.4^a^	0.01
MDA (nmol/mL)	5.13 ± 0.03	5.02±0.02	4.98 ± 0.01	4.94 ± 0.01	0.42
TAC (U/mL)	6.14 ± 0.12^b^	6.21 ± 0.11^ab^	6.27 ± 0.23^ab^	6.34 ± 0.18^a^	0.01

Small letters (^a-c^) on means in rows indicate significant difference at *P* < 0.05.

SOD: Superoxide dismutase.

GPx: glutathione peroxidase.

MDA: malondialdehyde.

TAC: total antioxidant capacity.

SS-0 (control), SS-10, SS-15 and Ss-20: Sesame seeds were supplemented at the rate of 10, 15 and 20 g/kg.

The effects of dietary sesame seed inclusion on intestinal microbial populations are summarized in Table 7. Salmonella counts were significantly reduced in all sesame-supplemented groups compared with the control (*p* = 0.02), with the lowest counts observed in the SS-20 group. A similar trend was observed for Escherichia coli, where higher levels of sesame seed resulted in significantly lower bacterial counts (*p* = 0.01).

**Table 7:** Impact of sesame seed supplemental diets on intestinal microflora (mean ± SEM) of laying hens (*n* = 5).

	SS-0	SS-10	SS-15	SS-20	P value
*Salmonella* (Log_10_ cfu/g)	6.08 ± 0.11^a^	5.76 ± 0.2^ab^	5.63±0.3^ab^	5.38 ± 0.11^b^	0.02
*E. coli* (Log_10_ cfu /g)	7.28 ± 0.3^a^	7.06 ± 0.5^ab^	6.79 ± 0.4^ab^	6.36 ± 0.4^b^	0.01

Small letters (^a-b^) on means in rows indicate significant difference at *P* < 0.05.

SS-0 (control), SS-10, SS-15 and Ss-20: Sesame seeds were supplemented at the rate of 10, 15 and 20 g/kg.

Intestinal morphometric measurements are presented in Table 8. Duodenal crypt depth differed significantly among treatments (*p* = 0.01), with shallower crypts observed in hens receiving sesame seed–based diets compared with the control. The villus-to-crypt ratio in the duodenum increased significantly with sesame seed inclusion (*p* = 0.02), particularly in the SS-15 and SS-20 groups. However, duodenal villus height was not significantly affected by dietary treatment (*p* > 0.05). In the jejunum, none of the measured morphological parameters showed significant differences among experimental groups (*p* > 0.05).

**Table 8:** Impact of sesame seed supplemental diets on intestinal morphology (mean ± SEM) of laying hens (*n* = 5).

	SS-0	SS-10	SS-15	SS-20	P value
**Duodenum**					
Villi Height (um)	382.35 ± 5.3	385.17 ± 6.7	388.67 ± 7.8	391.48 ± 8.9	0.30
Crypt depth (um)	34.2 ± 1.7^a^	34.36 ± 1.6^a^	33.42±1.2^ab^	33.46 ± 2.3^ab^	0.01
Villus crypt ratio	11.18 ± 2.6^b^	11.21 ± 4.3^b^	11.63 ± 1.7^a^	11.70 ± 2.9^a^	0.02
**Jejunum**					
Villi Height (um)	315.81 ± 5.7	317.01 ± 7.6	318.23 ± 5.4	320.4 ± 8.7	0.20
Crypt depth (um)	29.46 ± 3.4	29.38 ± 2.3	29.33 ± 1.5	29.29 ± 1.2	0.51
Villus crypt ratio	10.72 ± 0.4	10.79 ± 0.5	10.85 ± 0.4	10.94 ± 0.5	0.42

Small letters (^a-b^) on means in rows indicate significant difference at *P* < 0.05.

SS-0 (control), SS-10, SS-15 and Ss-20: Sesame seeds were supplemented at the rate of 10, 15 and 20 g/kg.

## Discussion

Dietary supplementation with bioactive feed additives has been widely recognized to enhance growth performance, nutrient utilization, physiological status, and product quality in poultry (Rahman et al., 2017; Hafeez et al., 2020; Ullah et al., 2022). In the present study, graded dietary supplementation of SS produced clear, dose-dependent improvements in productive performance, reflected by increased daily and weekly egg production, higher hen-day production, and improved FCR. These improvements occurred without adverse effects on feed intake, indicating enhanced nutrient efficiency, consistent with previous findings in layers and quail where sesame seeds or sesame oil improved egg number, egg mass, and FCR without altering feed consumption (Al-Daraji et al., 2012; 2013; Baghban-Kanani et al., 2019).

The observed enhancements in productivity are likely due to the high nutritional value of sesame seeds, including metabolizable energy, digestible protein, unsaturated fatty acids, and methionine-rich amino acids, which support yolk precursor synthesis and metabolic efficiency. Studies by Diarra et al. (2008) demonstrated that sesame seed meal can partially replace soybean meal as a methionine source, maintaining laying performance at moderate inclusion levels (≤12.5 %) while higher levels (>25 %) may compromise performance. Our findings confirm that graded inclusion up to 20 g/kg optimizes production without negative effects on feed intake or egg quality.

Lipid metabolism was positively modulated by sesame seed supplementation. Reductions in total and LDL cholesterol, alongside increased HDL cholesterol, can be attributed to bioactive lignans (sesamin, sesamolin), phytosterols, and phenolic compounds, which regulate hepatic HMG-CoA reductase and enhance bile acid synthesis (Baghban-Kanani et al., 2019; Al-Daraji et al., 2012; Onunkwo et al., 2015). These lipid-lowering effects are consistent with improvements in serum profiles reported in layers and quail fed sesame-based diets.

Although dietary SS supplementation resulted in statistically significant increases in antioxidant enzymes (SOD and GPx) and TAC, the absolute magnitude of these changes was relatively modest across treatments. Such incremental elevations, however, should be interpreted within a physiological and commercial framework rather than dismissed as negligible. In commercial poultry production, even small but consistent improvements in antioxidant status may contribute to enhanced oxidative stability, particularly under chronic or cumulative stress conditions. The absence of a significant reduction in MDA suggests that SS supplementation primarily enhanced endogenous enzymatic defense mechanisms rather than exerting an immediate effect on lipid peroxidation. This upregulation of antioxidant enzymes may serve a preventive role by increasing the birds’ resilience to oxidative challenges rather than producing large short-term biochemical shifts. These findings are consistent with previous reports indicating that sesame-derived lignans and phenolic compounds act as modulators of antioxidant enzyme activity rather than potent suppressors of oxidative damage markers (Wei et al., 2022; Yaseen et al., 2021; Baghban-Kanani et al., 2019). Therefore, while the observed differences are quantitatively small, their biological relevance lies in supporting long-term physiological robustness and sustainability of laying hens under commercial production conditions.

The antimicrobial potential of sesame seed was reflected by reductions in fecal *Salmonella* and *E. coli* counts, although the absolute magnitude of these reductions was relatively modest. Nevertheless, even small decreases in enteric pathogen load can be biologically meaningful under commercial poultry production systems, as they may reduce environmental contamination and cumulative infection pressure within flocks. These effects are likely associated with sesame-derived phenolic compounds, such as sesamol, and with improvements in mucosal antioxidant status that may enhance intestinal barrier integrity. Concurrent changes in intestinal morphology, including slightly increased villus-to-crypt ratios and shallower crypts in the duodenum, were also quantitatively small but directionally favorable. Such subtle structural adaptations are consistent with improved epithelial turnover efficiency and nutrient utilization rather than extensive morphological remodeling, which is typical in healthy birds fed adequate diets. In this context, these changes may have contributed to the observed improvement in FCR. Similar modest but functionally relevant improvements in gut structure and performance have been reported with processed sesame meal or roasted sesame hull supplementation in poultry diets (Mahmoud et al., 2015).

Egg quality traits were unaffected by dietary sesame supplementation, consistent with prior studies showing that sesame meal primarily influences yolk lipid metabolism rather than structural egg characteristics (Baghban-Kanani et al., 2019; Al-Daraji et al., 2013). This indicates that while sesame improves metabolic and physiological indices, it does not compromise external or internal egg quality at the tested inclusion levels.

Overall, the present study demonstrates that sesame seed supplementation up to 20 g/kg enhances productive performance, antioxidant status, serum lipid profile, gut microbial load, and intestinal morphology in Rhode Island Red laying hens without negative effects on feed intake or egg quality. Future studies employing molecular approaches, including 16S rRNA sequencing and lipidomic analyses, may provide mechanistic insights into gut microbial shifts, hepatic lipid regulation, and yolk composition. Additionally, longer-term trials would help determine whether extended supplementation further reduces lipid peroxidation and optimizes gut morphology.

## Conclusion

Partial replacement of soybean meal with sesame seed up to 20 g/kg enhanced productive performance, hen-day egg production, serum lipid profile, antioxidant status, and duodenal morphology in Rhode Island Red laying hens, while reducing pathogenic gut bacteria without affecting egg quality. The highest inclusion level (SS-20) yielded the greatest benefits, demonstrating the potential of sesame seeds as a natural, functional feed ingredient in layer diets. These results provide practical guidance for improving poultry nutrition and health; however, further research is needed to assess long-term effects, underlying molecular mechanisms, and performance under different management, environmental, or breed conditions.

## Ethical Approval

The Committee on Animal Rights and Welfare, The University of Agriculture Peshawar, Pakistan approved this study (FAHVS/122/2023).

## Data availability

Data will be made available from the authors upon reasonable request.

## CRediT authorship contribution statement

**Saba Ajam:** Methodology, Investigation. **Sar Zamin Khan:** Data curation, Conceptualization. **Hanan Al-Khalaifah:** Resources, Funding acquisition. **Saher Musrrat:** Validation, Writing – review & editing. **Rifat Ullah Khan:** Writing – review & editing, Writing – original draft. **Abdul Hafeez Abdul Razzaq:** Resources, Funding acquisition. **Ala Abudabos:** Writing – review & editing, Writing – original draft. **Ihteshamul Haq:** Validation, Visualization. **Ibrahim A. Alhidary:** Resources, Funding acquisition.

## Disclosures

Authors declare no conflict of interest. AI Chatgpt has been used for english language and text generation.
